# Sulphur Rendered Soft

**Published:** 1863-11

**Authors:** 


Sulphur Rendered Soft.—A young German chemist has discovered that, by the addition of a small quantity of iodine, pure sulphur is rendered perfectly soft, and may be formed into thin leaves as flexible as wax.

